# Reconstruction of 12-lead ECG: a review of algorithms

**DOI:** 10.3389/fphys.2025.1532284

**Published:** 2025-04-25

**Authors:** Ekenedirichukwu N. Obianom, G. André Ng, Xin Li

**Affiliations:** ^1^ Department of Cardiovascular Sciences, University of Leicester, Leicester, United Kingdom; ^2^ Department of Cardiology, University Hospitals of Leicester NHS Trust, Leicester, United Kingdom; ^3^ Leicester British Heart Foundation Centre of Research Excellence, Leicester, United Kingdom; ^4^ Leicester National Institute for Health and Care Research Biomedical Research Centre, Leicester, United Kingdom; ^5^ School of Engineering, University of Leicester, Leicester, United Kingdom

**Keywords:** ECG, reconstruction, neural networks, machine learning, review, regression

## Abstract

**Purpose:**

This paper aims to review the literature on 12-lead ECG reconstruction, highlight various algorithmic approaches and evaluate their predictive strengths. In addition, it investigates the implications of performing reconstruction in particular ways.

**Methods:**

This narrative review analysed 39 works on the reconstruction of 12-lead ECGs, focusing on the algorithms used for reconstruction and the results gotten from using these algorithms.

**Results:**

The works analysed featured the use of as little as one lead and as much as four leads for reconstruction of the other leads. Linear and nonlinear (including artificial intelligence) algorithms showed promising performances. Their outputs had correlations of greater than 0.90 depending on how the reconstruction models were built.

**Conclusion:**

Three leads are optimal as input predictors for minimal reconstruction errors, but there is no universal algorithm that applies to every reconstruction task. Both linear and nonlinear algorithms can achieve high correlations, and minimal root means square errors. Hence, planned steps are needed when deciding how to manipulate the data and build the models to achieve high accuracies.

## 1 Introduction

**FIGURE 1:**
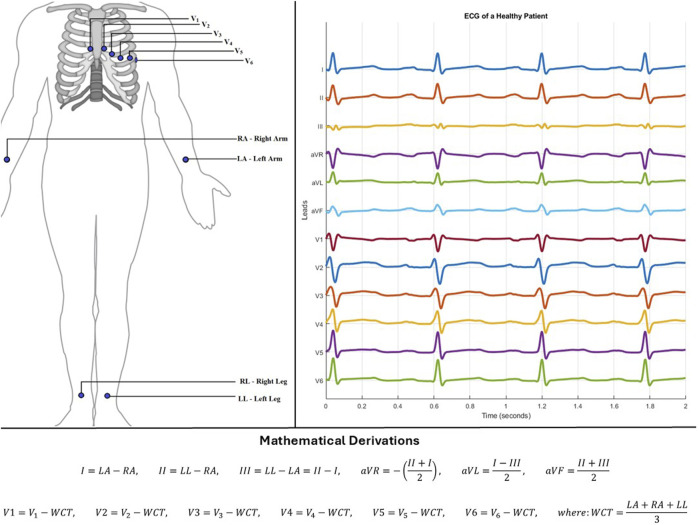
Left-Electrode positions for standard 12-lead system. Right-Lead output from a healthy patient using standard 12-lead system. Below–Mathematical derivation of each lead.

Electrocardiogram (ECG) is a non-invasive and painless method for quickly examining the electrical activity of the heart (Einthoven, 1912). The basic concept is for electrodes to be strategically placed on parts of a patient’s body to record the electrical activity of the heart. These electrodes are passive components; that is, they do not produce any form of electrical signal (Lewis, 1912), they only record the signals sensed. Over the years, there has been research into various electrode positions to acquire the best information in the simplest form (Frank, 1956; Mason and Likar, 1966; Dower et al., 1988). Some of the common systems developed include the Mason-Likar lead system (Mason and Likar, 1966), the Frank Vectorcardiogram (VCG) lead system (Frank, 1954), and the EASI lead system (Dower et al., 1988). Amidst these systems, a standard 12-lead system (S12) has been agreed upon (Wilson et al., 1954) (Figure 1). This standard has shaped the nature of electrocardiography ever since and is the commonly used method for diagnosis (AlGhatrif and Lindsay, 2012).

The S12 leads (limb leads - I, II, III, aVR, aVL, aVF and precordial/chest leads – V1, V2, V3, V4, V5, V6) are generated by a mathematical combination of 10 electrodes. Since these electrodes are passive, they record noise artefacts in addition to heart signals. These artefacts can come from the movement of patients during recording, wrong placement of electrodes, electrical interference, or even no-contact between the electrode and the patient’s skin. This has led researchers to consider alternative methods to acquire S12 with fewer electrodes. This can help to recover missing or noisy leads, or even reduce the number of electrodes needed for recording. Moreso, this can help with patients who need to be monitored outside the hospital.

In line with this, devices such as the Holter monitor (Holter, 1961), patch devices, and smart watches have been designed to acquire heart data with minimal contact with the human body. However, they do not provide as much information as S12. This has led researchers to focus on predicting S12 leads with fewer leads. For example, two leads can be used to predict the remaining ten leads (Vemishetty et al., 2019). Over time, many algorithms ranging from linear to non-linear algorithms have been developed for this purpose. These have included the use of linear regression, principal component analysis, independent component analysis, and even neural networks, to create patient-specific or generic models. The patient-specific models aimed at creating algorithms specific to a patient (Schreck et al., 1998), while the generic models aimed at building algorithms that could be used for any patient irrespective of age, race, gender or ailment. None of these algorithms have a perfect reconstruction capability and all have their strengths and drawbacks. It is noteworthy that the inability to perfectly reconstruct ECG is partly due to the complexity, diversity and everchanging nature of the heart (especially with the generic models).

The differences in algorithm capabilities have inspired this work. This paper aims to narratively review the literature on 12-lead ECG reconstruction, highlight various algorithmic approaches and evaluate their predictive strengths. In addition, it investigates the implications of performing reconstruction in particular ways.

The publications chosen for this review were selected from various databases including Institute of Electrical and Electronics Engineers (IEEE), Medline, Scopus, Web of Science, and The Cochrane Library. The time frame chosen for this review was from the year 1980–2023 (spanning 43 years), to capture the traditional methods that were used for ECG reconstruction and the transition into the more recent algorithms that have been adopted. The inclusion criteria for the selected publications were all publications in English language, using either simple mathematical or machine learning methods, and were journal articles or conference papers. The keywords used for the search queries were:

(ECG OR Electrocardiogra* OR EKG) AND (Reconstruct* OR recover* OR regenerat* OR extrapolat* OR retriev* OR reduc* OR predict* OR calculat* OR estimat* OR approximat* OR comput*) AND (12 lead OR twelve lead OR electrode OR reduced electrode).

## 2 Lead importance in reconstruction

To reconstruct S12, it is paramount to know the leads that provide the best extrapolatory significance. Using factor analysis, Schreck et al. (1998) aimed to find the smallest set of leads necessary to describe S12. They derived 12 eigenvalues that had a magnitude that correlated to the significance of its eigenvector. Since leads III, aVR, aVL, and aVF could be calculated from leads I and II, they were not used in this analysis. A patient-specific transformation matrix was obtained for this analysis, and the predictive power was tested with varying numbers of leads. They found that using three leads to reconstruct the other leads accounted for most of the information content in S12 (99.12% ± 0.92%). Using more than three leads added very little information to the reconstruction.

Nelwan et al. (2000), in line with Scherer et al. (1989), recommended the use of I, II and V2 (4 electrodes) for reconstruction. A substantial decrease in the correlation between the reconstructed S12 (RS12) and the original S12 was observed when three leads and four leads were used as predictors. The decrease was from an average of 0.994 to 0.983 in the patient-specific models and from 0.963 to 0.926 in the generic models. Nelwan et al. (2004a) proposed that a dependable patient-specific model can be built with I, II and V2, but when a generic model is designed, V5 should be included (5 electrodes). To substantiate the reconstructive power of these four leads, Nelwan et al. (2007) assessed the root mean squared error (RMSE) of the RS12 of four lead systems at the time (Dower et al., 1988; Nelwan et al., 2000). They found that I, II, V2, and V5 had the lowest RMSE. Nelwan et al. (2008a) revisited the significance of various frontal leads in reconstruction and found that, although minute, combination of limb leads other than I and II may improve the results based on the lead being predicted.

However, Wang et al. (2005) argued that only two precordial leads were enough to reconstruct S12. By generating a coefficient matrix using two precordial leads, they reproduced other leads with a minimum coefficient of 0.954. Although there were no specific leads that could reconstruct all leads, it showed that two leads were capable of reliable reconstruction in lead-specific cases. Matyschik et al. (2020) supported this finding when they used only one precordial lead to reconstruct S12 with the help of a more complex patient-specific model. They used a variational autoencoder decoder (VAE) combined with a convolutional neural network (CNN) and either V2, V3, or V4 to reconstruct S12 depending on the patient in question. With the advancement of computing power and the development in machine learning (ML), one precordial lead could be enough to provide a reliable RS12 (Matyschik et al., 2020).

Butchy et al. (2023) decided to determine the lead importance by comparing the correlation between each lead. They found that limb leads were highly correlated with themselves and with V1, V4, V5, and V6. V2 and V3 were more strongly correlated with themselves than with other leads. By comparing the $R^{2}$ of the reconstruction of other leads with or without each of the electrodes, it was found that RA, LA, LL, and $V_{3}$ had the worst outcomes when excluded as inputs and the best outcomes when included as inputs. This, in their opinion, points to the importance of the limb leads and V3 as the most important leads for reconstruction of S12.

Furthermore, the misplacement of electrodes by physicians due to various reasons requires attention. This causes incorrect signals to be recorded and inadvertently cause incorrect models to be created or poor reconstructions from the available models. Finlay et al. (2009) reported that reconstruction errors become evident when electrodes are placed at least 15 mm from the correct positions. This was when I, II, V2, and V5 were used for reconstruction. In contrast, when EASI electrodes were used for reconstruction, significant errors were not evident until 5 cm from the correct electrode position.

Nonetheless, it is easy to fall into the assumption that there should be a single perfect lead set to reconstruct the remaining S12 leads. It is paramount to consider the complexity of the reconstruction situation (Feild et al., 2008). When choosing the lead set it is important to consider the lead that needs to be reconstructed. Each lead has an optimal lead combination unique to them for the best reconstruction (Yoo et al., 2023). Additionally, choosing a lead set that is in the region that needs to be reconstructed would yield better results than a generic lead set.

## 3 Algorithms for reconstruction

In the 39 studies reviewed in this paper, reconstruction algorithms are broadly categorized into linear and non-linear types to draw on the different ways in which these algorithms have been used and their limitations. To assess the performance of these algorithms, the studies employed various metrics such as correlation coefficient, similarity coefficient, mean squared error (MSE), RMSE and r-squared ( $R^{2}$ ), though not all were used simultaneously in each study. These metrics provide a basis for evaluating how well the algorithms perform in reconstruction. Correlation defines how well the RS12 changes with respect to the original S12, while RMSE defines the average error over time. These become important when considering the shape of segments of the signal. Additionally, it is important to recognise that the type of model affects the performance of the model. A patient-specific model usually performs better than a generic model. Depending on the application, if a patient-specific model can be designed, it is advisable to do so.

### 3.1 Linear algorithm

This type of algorithm follows the assumption that body surface potentials are linearly related to each other. Mathematically speaking:

$$y=c_{1}x_{1}+c_{2}x_{2}+\ldots +c_{n}x_{n}+d$$

where:y=the potential  to  be  reconstructed

$$c_{1},c_{2},\ldots ,c_{n}=constant\;\;coefficients$$

$$x_{1},x_{2},\ldots ,x_{n}=the\;\;available\;\;body\;\;surface\;\;potentials$$

d=constant

Dower et al. (1988) approached the reconstruction with a spatial transform. The vector loops were rotated prior to being transformed. They showed that this algorithm works especially well when extrapolating from VCG. They used previously published generic constants (Dower et al., 1980) to predict S12. Nelwan et al. (2001) argued otherwise. They claim that the basic linear regression, without spatial transform, was enough to give reliable results. This claim was supported by comparing the reconstruction correlation of both algorithm on the same patient sets. Linear regression performed better than using inverse Dower transformation.

Scherer et al. (1989) used a patient-specific linear model to reconstruct V1, V3, V4, V5, and V6 from I, II and V2. They found that a trained model could be used for three consecutive days with a correlation greater than 0.96 on all leads. There was also good similarity between RS12 and S12, with a consistent p value greater than 0.5 (Scherer and Willems, 1992; Maheshwari et al., 2013). Vemishetty et al. (2019) deviated slightly in how they used the same leads for reconstruction. They used I and V2 to reconstruct II and then used I, V2 and reconstructed II to reconstruct the other leads. The average $R^{2}$ and correlation coefficient of the RS12 for healthy patients were 0.919 and 0.957, respectively, for healthy patients but decreased with bundle branch block and myocardial infarction. Least square (LS) fit and Heart-Vector Projection (HVP), which are common transformation matrix generation methods were used. Other matrix transform generation tools that make use of singular value decomposition (SVD) have also shown promising results (Padhy and Dandapat, 2016).

There is also the assertion that generic algorithms perform worse than patient-specific algorithms, but this does not mean the generic models are not useful (Nelwan et al., 2004b; Nelwan et al., 2004c; Nelwan et al., 2008b). It was found that the two approaches were capable of reconstructing S12 with a high degree of accuracy from leads I, II, V2 and V5 while using a linear transformation matrix. The RS12 could be used to predict certain heart conditions (Nelwan et al., 2004c; Guldenring et al., 2012). Xue (2007) showed that RS12 predicted from leads I, II, V1 and V5 could be used an ML model to classify ischemia patients. Their RS12 was gotten using linear regression equations for each lead.

Various combinations of leads have also been used to predict S12. Horácek et al. (2008) compared the predictive capability of all the possible combinations (15) of two precordial leads from both standard 12-lead ECG and Mason-Likar 12-lead ECG. These leads, with I and II, were used to predict other leads. Although V2 and V4 had the best correlation, 14 of the 15 lead sets performed with a mean similarity coefficient above 99%. The average RMSE was also consistently less than 52 mV.

Many researchers have also explored unconventional electrode placements. RS12 from these systems is also capable of predicting (with a high level of accuracy) health conditions (Babic et al., 2023). Hadzievski et al. (2004) proved a that linear transformation matrix was sufficient to transform unconventional potential recordings into S12 (Vukajlovic et al., 2010; Vukcevic et al., 2010; Vukajlovic et al., 2011; Ivanovic et al., 2019; Grande-Fidalgo et al., 2021). They found that 80.2% of the RS12 were identical to S12 without visible discrepancy. Additionally, this lead system allows for a single patient-specific transformation matrix to be used reliably for reconstruction for up to 6 months (Gussak et al., 2012).

Some researchers have approached reconstruction by piling different stages of linear equations. A snippet of this can be seen in the work of Vemishetty et al. (2019), where they had to reconstruct II and use it to reconstruct other leads. The piling strategy developed by Burnes et al. (1998) is called inverse-forward interpolation. This algorithm involves inversely reconstructing an arbitrary interior surface from body potentials, then using a forward model to reconstruct S12. Bear et al. (2017) used this algorithm to interpolate 252 body surface potentials (BSPMs) to reconstruct S12. Their result showed correlations greater than 0.88 across leads between RS12 (of a generic model) and S12.

Independent component analysis (ICA) is another methodology (Ostertag and Tsouri, 2011; Tsouri and Ostertag, 2014). This algorithm involves finding independent features of the signal that can be used to reconstruct them in future signals. The complexity here is that a single output is assumed to be the product of a mixing matrix and an independent component matrix, which are both unknown to researchers. The best way to find these unknowns is to start with an initial guess and then keep changing their values, smartly, until they converge based on the training data. A problem faced is deciding the number of independent components that these variables should have. Depending on the leads used as input and the number of independent components used, correlations greater than 0.95 can be achieved (Ostertag and Tsouri, 2011; Tsouri and Ostertag, 2014).

A similar algorithm is the heart-dipole model (Sindreu et al., 2023). This model assumes that each body surface potential is a linear combination of three-dipole components of the heart. This implies that at any given time, the electric field in the body is in equilibrium with its source in the heart. If these dipole components can be found in conjunction with the lead vectors, any given lead can be derived. Sindreu et al. (2023) reported that a patient-specific heart-dipole model was able to produce RS12 with a 0.966 correlation to S12.

Another important approach was the use of state-space modelling by Lee et al. (2016). This model can capture nonlinear properties of signals. It is also able to predict real-time events of the output signals with respect to the input signals. They found that this model performed better than linear transform models. It had a greater mean correlation (0.937 compared to 0.843) and lower mean RMSE (86.33 µV compared to 128.17 µV).

The use of linear models seems promising. However, Gregg et al. (2008) shed more light on the down sides of this model. In accordance with the suggestion of Feild et al. (2008), Gregg et al. (2008) expressed how this model failed when dealing with multiple heart conditions. That is, coefficients deduced during optimal operation of the heart may not stand during abnormal readings (Vemishetty et al., 2019; Guldenring et al., 2012). It is either various models are designed for various heart conditions, or a more complex model is designed to accommodate these conditions. A summary of the linear approaches examined in this paper is shown in Table 1.

**TABLE 1:** Summary of Linear Approaches taken in the Reconstruction of ECGs including the kind of algorithm adopted, the input leads used, either they used generic models (True), patient-specific models (False), or analysed both (Both), the average correlation (r), and the source of the dataset (the name of the source, private database, or personally acquired).

S/N	Paper	Input	Algorithm	Generic model	r	Dataset
1	Dower et al.(1980)	Frank	Spatial transform	True	-	Personal
2	Dower et al. (1988)	EASI	Spatial transform	True	-	Personal
3	Scherer et al. (1989)	I, II, V2	Linear transformation	False	0.988	Personal
4	Nelwan et al. (2004c)	I, II, V2, V5	Linear transformation	Both	-	REPAIR
5	Maheshwari et al. (2013)	I, II, V2	Linear transformation	False	0.982	INCART (Goldberger et al., 2000) and PTBDB (Goldberger et al., 2000; Bousseljot et al., 1995)
6	Padhy and Dandapat (2016)	I, II, V5	Linear transformation SVD	False	0.947	PTBDB (Goldberger et al., 2000; Bousseljot et al., 1995)
7	Vemishetty et al. (2019)	I, V2	Linear transformation LS HVP	False	0.957	PTBDB (Goldberger et al., 2000; Bousseljot et al., 1995)
8	Guldenring et al. (2012)	I, II, V2, V5	Linear transformation	Both	-	Personal
9	Xue (2007)	I, II, V1, V5	Linear regression	True	-	Private
10	Horácek et al. (2008)	I, II, and 2 posterior leads	Linear transformation	Both	-	Personal
11	Babic et al. (2023)	5 electrode system	Linear transformation	Both	-	Personal
12	Hadzievski et al. (2004)	5 electrode system	Linear transformation	False	-	Personal
13	Vukajlovic et al. (2010)	5 electrode system	Linear transformation	False	-	Personal
14	Vukcevic et al. (2010)	5 electrode system	Linear transformation	False	-	Personal
15	Gussak et al. (2012)	5 electrode system	Linear transformation	False	-	Personal
16	Bear et al. (2017)	252 BSPM	Inverse-forward interpolation	True	>0.880	Simulated and 6 patients
17	Ostertag and Tsouri (2011)	V2, V5	ICA	False	0.955	PTBDB (Goldberger et al., 2000; Bousseljot et al., 1995)
18	Tsouri and Ostertag (2014)	I, II, V2 vs. Frank	ICA	False	0.980	PTBDB (Goldberger et al., 2000; Bousseljot et al., 1995)
19	Sindreu et al. (2023)	I, II, and 2 posterior leads	Heart dipole model	False	0.966	Personal
20	Lee et al. (2016)	I, II, III	State space model	False	0.992	PTBDB (Goldberger et al., 2000; Bousseljot et al., 1995)

### 3.2 Nonlinear algorithm

Unlike the latter, this type of algorithm recognises that the relationship between leads of S12 could be more complex than what simple linear transformations can explain due to artefacts that are recorded alongside and variations in lead locations. As many researchers have suggested, a more complex algorithm could improve the likelihood of a more reliable reconstruction (Vemishetty et al., 2019; Feild et al., 2008; Guldenring et al., 2012; Lee et al., 2016; Gregg et al., 2008).

With the increase in computing power, more complex statistical models have begun to appear in the last 2 decades. One of such models is the support vector machine (SVM) model. This model maps the input data into a higher dimension and then estimates a function capable of reconstructing the output data. Using a large dataset, this model can be trained to effectively reconstruct S12. Yodjaiphet et al. (2012) reconstructed V2, V3, V4, and V5 using SVM, with inputs I, II, V1, and V6. They trained this model on 14 patients and obtained an RMSE of less than 0.29 mV.

#### 3.2.1 Neural networks

Artificial Neural Networks (ANNs) are among the more complex and nonlinear models used today. Variations in ANNs exist, but the basic concept of connecting inputs to various decision-making neurons remains the same. A common ANN is the feed-forward network (FFN). This network has no feedback, such that it makes predictions based on only the present input given. Atoui et al. (2004) showed that a patient-specific model built with the FFN, using I, II and V2 as inputs, outperformed the linear transformation algorithm. Some years later, they showed that scrutiny of the input data can improve the outcome of the network (Atoui et al., 2010). They improved the correlation of RS12 from median of 0.957 in patient-specific linear transform model to median of 0.975 in patient-specific FFN model. This model has also been shown to work with patch electrodes to predict RS12 with a median correlation of 0.92 (Lee et al., 2020).

Chen et al. (2015) built a similar patient-specific network but used the combination of genetic algorithm (GA) and back propagation (BP) training techniques to achieve better results than using only BP for training which produced a mean correlation of 0.948. Xu et al. (2018) argued that training an FFN with a general vector machine (GVM) creates a more stable and reliable algorithm. Their model achieved correlations higher than 0.81 (compared to the GA-BP correlation of as low as 0.79) on their dataset. The GVM is a methodology built on the Monte Carlo algorithm. It is useful when the dataset is very large, as in the S12 reconstruction. However, in contrast to Feild et al. (2008) and Atoui et al. (2004), Xu et al. (2021) claimed that linear regression outperforms FFN with an average correlation of 0.901 compared to 0.879. The inputs used were I, II, and V1.

Mulyadi and Supriyanto, (2019) introduced a novel approach to how the FFN was used. Since the ECG is a combination of different repolarisation and depolarisation segments, these segments should be derived separately and fused together. Using the EASI electrodes as input, they showed that this segment-specific reconstruction had a greater correlation than the full cycle reconstructions with an average RMSE reduction of 65.17% on all leads.

Long short-term memory (LSTM) is another methodology in ANNs used for time-series data. Unlike the FFN, the predictions are made based on both the present and past inputs. By predicting V1, V3, V4, V5, and V6 from I, II, and V2, average correlations of 0.95 in generic models can be attained with this algorithm (Zhang and Frick, 2019; Dhahri et al., 2022; Kapfo et al., 2022). Patch electrodes have also been shown to produce correlations greater than 0.92 on all leads with the patient-specific model of this algorithm (Sohn et al., 2020). Using other time-series focused networks and changing the number of inputs could also be highly important. Smith et al. (2021) showed that using all limb leads, and V2 as inputs and a focused time-delay neural network (FTDNN), correlations higher than 0.861 could be achieved. It is important to note that the work of Smith et al. (2021) still uses 4 electrodes, similar to other researchers, but with seven leads as compared to the three leads of most.

A widely known ANN method used for image recognition is CNNs. Panda et al. (2014) showed that this can be applied in reconstruction. They built a patient-specific model which could predict V1, V3, V4, V5, and V6 from I, II, and V2 with an $R^{2}$ greater than 0.71. This algorithm is well supported by Wang et al. (2019), who achieved correlations greater than 0.938 on a database with 290 patients with a generic model.

The autoencoder-decoder U-Net ANN has also been used for S12 reconstruction. It is a complex ANN that compresses the input and decompresses it while comparing it to the expected output. Combining a generative adversarial network (GAN) and a U-Net, Yoon et al. (2022) built an ANN with all limb leads as input. They had a mean MSE of 0.038. Expanding the capabilities of ML, Beco et al. (2022) showed that S12 could be predicted with only lead II. They also used an autoencoder-decoder U-Net ANN to do this. They obtained promising results, but they also showed that lead II did not perform particularly well in predicting aVR, aVL, and aVF. Other single lead input U-Net architecture have been explored and have shown better results in predicting aVR, aVL, and aVF (Garg et al., 2023). Gundlapalle and Acharyya, (2022) also used lead II but used a different network architecture. Their method involved creating various generic models that combined CNN and LSTM to reconstruct various leads. Outstanding mean values of 0.936 $R^{2}$ and 0.973 correlation was achieved. A summary of the nonlinear approaches examined in this paper is shown in Table 2.

**TABLE 2:** Summary of Nonlinear Approaches taken in the Reconstruction of ECGs including the kind of algorithm adopted, the input leads used, either they used generic models (True), patient-specific models (False), or analysed both (Both), the average correlation (r), and the source of the dataset (the name of the source, private database, or personally acquired).

S/N	Paper	Input	Algorithm	Generic model	r	Dataset
1	Yodjaiphet et al. (2012)	I, II, V1, V6	SVM	True	-	PhysioNet (Moody et al., 2000)
2	Atoui et al. (2004)	DI, DII, V2	FFN	False	0.974	Personal
3	Atoui et al. (2010)	I, II, V2	FFN	Both	0.930/0.962	Personal
4	Lee et al. (2020)	3 lead 4 electrode patch device	FFN	True	0.893	Personal
5	Chen et al. (2015)	I, II, V2	FFN	False	0.948	PTBDB (Goldberger et al., 2000; Bousseljot et al., 1995)
6	Xu et al. (2018)	I, II, V2	FFN	True	0.892	PTBDB (Goldberger et al., 2000; Bousseljot et al., 1995)
7	Xu et al. (2021)	I, II, V1	FFN	True	0.879	PTBDB (Goldberger et al., 2000; Bousseljot et al., 1995)
8	Mulyadi and Supriyanto (2019)	EASI (4)	FFN	False	0.999	PTBDB (Goldberger et al., 2000; Bousseljot et al., 1995)
9	Zhang and Frick (2019)	I, II, V2	LSTM	False	0.830	MITBIH (Goldberger et al., 2000; Moody and Mark, 2001) and PTBDB (Goldberger et al., 2000; Bousseljot et al., 1995)
10	Dhahri et al. (2022)	I, II, V2	LSTM	True	0.950	PTBDB (Goldberger et al., 2000; Bousseljot et al., 1995)
11	Kapfo et al. (2022)	I, II, V2	LSTM	False	0.980	PTBDB (Goldberger et al., 2000; Bousseljot et al., 1995)
12	Sohn et al. (2020)	3 lead 4 electrode patch device	LSTM	False	0.949	Personal
13	Smith et al. (2021)	Limb leads and V2	FTDNN	True	0.903	PTBDB (Goldberger et al., 2000; Bousseljot et al., 1995)
14	Panda et al. (2014)	I, II, V2	CNN	False	-	PTBDB (Goldberger et al., 2000; Bousseljot et al., 1995)
15	Wang et al. (2019)	I, II, V2	CNN	True	0.950	PTBDB (Goldberger et al., 2000; Bousseljot et al., 1995)
16	Yoon et al. (2022)	Limb leads	GAN and U-Net	True	-	China DB (Zheng et al., 2020) and PTBXL (Goldberger et al., 2000; Wagner et al., 2020)
17	Beco et al. (2022)	II	Autoencoder decoder	True	0.672	INCART (Goldberger et al., 2000) and PTB-XL (Goldberger et al., 2000; Wagner et al., 2020)
18	Garg et al. (2023)	II	U-Net	True	0.805	PTBXL (Goldberger et al., 2000; Wagner et al., 2020)
19	Gundlapalle and Acharyya (2022)	II	LSTM-CNN	True	0.973	PTBDB (Goldberger et al., 2000; Bousseljot et al., 1995)

## 4 Discussion

Reconstruction of standard ECG leads has been a major consideration in the field of electrocardiography, aimed at reducing the number of electrodes and recovering missing leads. Feild et al. (2008) outlined a step-by-step procedure of things for this purpose and several key points need to be considered based on related studies seen in this paper. Firstly, the decision of the best input lead set is critical. As seen from all previous works, this affects the model used for transformation. Every model is built with respect to what the inputs and outputs will be. Most models, irrespective of the input leads, demonstrate promising performance based on the nature and size of the data used to build the model. Despite this, the most common input leads that have been used were proposed by Nelwan et al. (2000), in line with Scherer et al. (1989). They recommended the use of I, II and V2 (4 electrodes) for the reconstruction of V1, V3, V4, V5, and V6.

In contrast, Butchy et al. (2023) reported that the best input leads were I, II and V3 (4 electrodes). They found this by comparing the correlation between each lead. They found that limb leads were highly correlated with themselves and with V1, V4, V5, and V6, while V2 and V3 were more strongly correlated with each other than with other leads. Clearly, three leads (including both limb leads, and chest leads) are optimal for the reconstruction. Nevertheless, the model built also plays a large role in determining the accuracy of the reconstruction.

Secondly, a major question that many researchers overlooked is the long-term reliability of their models. The heart is an everchanging organ, it is important to consider how often a new model needs to be designed. Fortunately, a few researchers have commented on this. A model, either generic or patient-specific, can be used for a few days to a few months without notable performance degradation (Scherer et al., 1989; Gussak et al., 2012; Maheshwari et al., 2016). However, more research needs to be conducted to ascertain the timewise reliability of any given model.

The kind of data used to train a model is also important. Though it has been shown that unprocessed ECG can be used as input data with good reconstructions (Obianom et al., 2024), models tend to perform worse in patients with cardiac disorders, than in healthy patients (Vemishetty et al., 2019; Feild et al., 2008; Guldenring et al., 2012; Lee et al., 2016; Gregg et al., 2008). Therefore, the kind of data used during the training of a model is essential to determining the performance of the model. While building a model, a researcher could consider using data from many patients with various heart conditions. This would require a large amount of patient data (thousands) to ensure that the right transforms are made. Conversely, a researcher can consider using data from a particular heart condition group. This would be a population-specific model and would not require as many patients.

More emphasis should also be drawn to the nature of the model. As much as the patient-specific models tend to always have better results, there are cases where the generic or population-specific models might be of great importance (Nelwan et al., 2004b; Nelwan et al., 2004c; Nelwan et al., 2008b). They can be especially useful in cases where quick information needs to be acquired. They can also be used to aid in the prediction of certain heart conditions (Nelwan et al., 2004c; Guldenring et al., 2012). Xue (2007) showed that RS12 predicted from leads I, II, V1 and V5 could be used to train an ML model to classify ischemia patients. Regarding the influence of datasets the model’s nature, any dataset can be employed to develop either a patient-specific or a generic model. For instance, the publicly available PTB database (Goldberger et al., 2000; Bousseljot et al., 1995) have been used to construct both types of models (Table 1, 2). The key takeaway is that patient-specific models typically produce more accurate results because no two human hearts are identical, and a patient’s own heart is the most precise model for itself.

There is the argument of nonlinear models being more effective than linear models in the reconstruction of S12. Many researchers have considered that the complexity of human physiology cannot be summed up in simple linear transforms (Feild et al., 2008). Feild et al. (2008) stresses that the complexity of the models needs to increase to models like Matyschik et al. (2020) and Yoo et al. (2023), beyond the linear transformation models (Dower et al., 1988; Scherer et al., 1989; Scherer and Willems, 1992; Nelwan et al., 2004c). This is attributed to significant limitations of linear models, such as their inability to account for noise artifacts. Counter to that, Xu et al. (2021) claims that linear regression performs better than FFN. Clearly, it is not about the complexity of the equations used in reconstruction, it is about how the equations were used for reconstruction (Mulyadi and Supriyanto, 2019). It is also worth noting that in device development and deployment, neural networks tend to require larger computing power, larger memory size, and have longer building time than classical algorithms. Therefore, choosing a nonlinear algorithm has its down sides particularly in device development.

Smith et al. (2021) also showed that using leads I, II, III, aVR, aVL, aVF, and V2 as inputs, correlations higher than 0.861 could be achieved. Although this might increase the processing power and the complexity of the models being used, this is worth considering. Like the popular I, II, and V2 inputs, these inputs still consist of four electrodes. Butchy et al. (2023) also noted that limb leads are highly correlated with V1, V4, V5, and V6. Therefore, this may provide a greater chance of better accuracy in reconstruction. Additionally, it is worth noting that various leads have stronger correlations with specific leads (Yoo et al., 2023). Therefore, providing all available leads as input may enable the models to give various weight to the necessary leads for reconstructing specific leads rather than relying on a minute lead set for the reconstruction of all leads.

It would also be valuable to analyse the performance of the models reviewed in this paper under a standardised framework. However, it is regrettable that the studies referenced in this paper utilised different databases and inconsistent performance metrics. Additionally, those that employed the same database may have not utilised the complete dataset provided. Nonetheless, the PTB database was predominantly used across various algorithms, input lead combinations, and both patient-specific and population-specific models, and can offer valuable insights (Table 1, 2). The trends discussed throughout this section are evident within these studies; patient-specific models generally yield better results, the nature of the training data affects the model performance, models performed worse with arrhythmic ECG than with rhythmic ECG, and input leads are very key to achieving accurate results.

Finally, the reconstruction of leads is clinically relevant in areas such as cardiac disease prediction systems. Since S12 has considerable redundant information, a model could be built to focus on reconstructing specific information in other leads which can be used as input for prediction models (Nelwan et al., 2004c; Guldenring et al., 2012; Xue, 2007). Furthermore, since the nature of data influences the reconstruction, this factor can be leveraged to reconstruct arrhythmic ECG signals. For instance, Gundlapalle and Acharyya (2022) and Vemishetty et al. (2019) trained models with only myocardial infarction patients and achieved an average correlation of 0.973 (population-specific approach) and 0.889 (patient-specific approach) respectively across all reconstructed leads. This demonstrates that models can reliably reconstruct arrhythmic ECG signals when trained on such data; however, their generalizability remains limited, as a single model has yet to demonstrate efficient reconstruction of both rhythmic and arrhythmic ECG signals (Feild et al., 2008; Guldenring et al., 2012; Lee et al., 2016; Gregg et al., 2008). For clinical applications, there is a need for either a sophisticated ensemble of models tailored to specific ECG rhythm types or a more advanced, unified model capable of reconstructing a broad spectrum of ECG patterns. ECG reconstruction is also highly relevant in the context of telemedicine. Given that most wearable devices have limited number of leads, these models can be used to reconstruct other leads from the recorded leads either on-site or via cloud processing. This enhances the availability of critical ECG information for physicians and prediction models to make accurate diagnosis (Babic et al., 2023; Gussak et al., 2012; Kapfo et al., 2022; Sohn et al., 2020; Panda et al., 2014). Moreover, it has been proven that these reconstruction models remain reliable from a few days to several months. As a result, they can be updated automatically or with scheduled checks to prevent performance degradation.

## 5 Conclusion

This paper considered the varying algorithms employed for the reconstruction of ECG from a subset of leads. ECG reconstruction is necessary for a variety of reasons. They are important for out-of-hospital monitoring, where patients are able enough to be discharged but constant monitoring is still needed. They are also important in emergency monitoring, where quick and minor decisions need to be made and can be used for disease prediction to gain quick insight into the plausible conditions of the heart. However, the accuracy of the reconstructions currently varies due to the different perspectives being taken.

**FIGURE 2:**
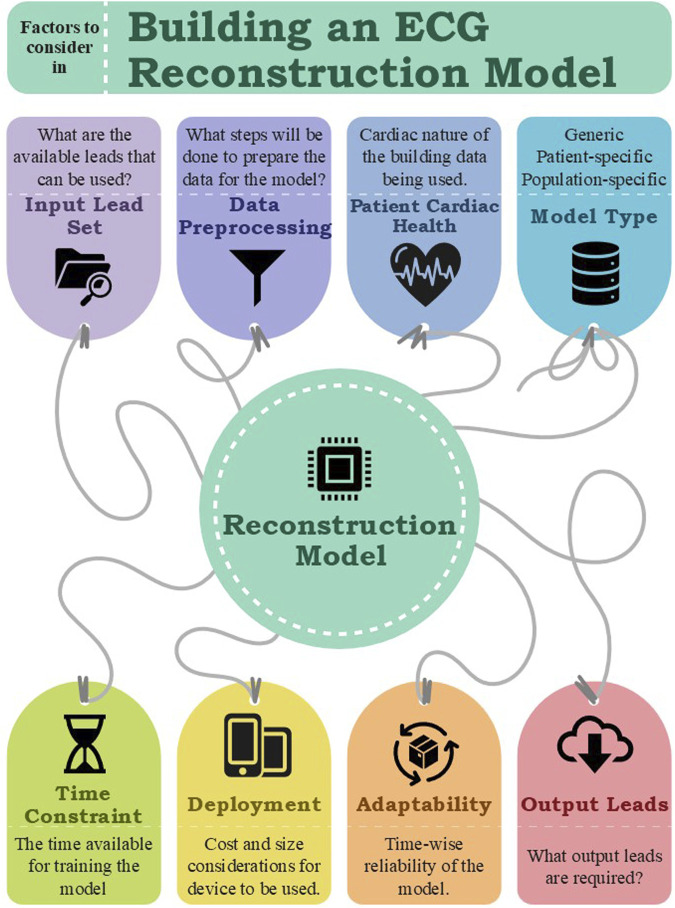
Factors to consider while building an ECG reconstruction Model.

This review identified that no single algorithm consistently outperforms others. To develop a reliable reconstruction model, careful consideration must be given to the selection of input leads, model architecture, the characteristics of the training data (particularly the patients’ heart conditions) and the intended purpose of the reconstruction. Following a structured and systematic approach in model development and data processing is essential to achieving high accuracy (Figure 2). These factors collectively contribute to the effectiveness and reliability of the reconstruction process and model.
